# Expectations of learner nurses in sampled clinical areas of the Limpopo province, South Africa

**DOI:** 10.4102/hsag.v27i0.2012

**Published:** 2022-11-08

**Authors:** Julia L. Mafumo, Mutshinyalo L. Netshikweta

**Affiliations:** 1 Department of Advanced Nursing Science, Faculty of Health Sciences, University of Venda, Thohoyandou, South Africa

**Keywords:** expectations, opinions, clinical learning areas, learner nurses, professional nurses, clinical placement

## Abstract

**Background:**

Nurse training in South Africa requires placing learner nurses in real-life setting for them to gain practical experience. To complete their training and be competent practitioners, learner nurses must have expectations and aspirations when they go for practice.

**Aim:**

This study sought to understand learner nurses’ expectations at clinical learning areas during placement.

**Setting:**

Four clinical learning areas in the Limpopo province were purposely sampled. Tertiary, regional and district hospitals were sampled to obtain information from different levels of care.

**Methods:**

A qualitative, descriptive and contextual design was used to explore the expectations of learner nurses during clinical placements. Permission to conduct the study was obtained from various bodies and ethical considerations were ensured. Nonprobability purposive sampling was used, and six focus groups were formed. Each focus group discussion (FGD) had six to eight participants. The FGDs were according to level of study, with the third and fourth levels each having two FGDs because of the number of participants.

**Results:**

The following three themes emerged: milieu in the clinical learning areas, learning in the clinical areas and self-actualisation.

**Conclusion:**

The clinical learning areas are institutions of promoting learning through practice. They should promote learning and offer support to learners so that learners meet their expectations, which may prevent discouragement and attrition.

**Contribution:**

This study adds to the body of knowledge in nursing education and practice because when students’ expectations are met, their training outcome might be positive leading to competent professional nurses.

## Introduction

Levett-Jones, Reid-Searl and Bourgeois (2018) observe that clinical placements are the health setting where the world of nursing comes to life and learner nurses learn how to think, feel and behave, in what they value and communicate while providing care. These clinical placements are mandatory in the training of learner nurses and for applying theory into practice, leading to the development of competency in the nursing profession. The clinical placement learning experience provides learners with the opportunity to interact with patients and community members, ensuring that clinical skills are obtained, underpinned by a sound knowledge base (Adjei et al. 2018). Previous studies show that health sciences students consider clinical placements to be a source of meaningful learning which directly impacts their professional development (Araya et al. 2018; Pryjmachuk et al. 2019; Soler et al. 2021). It is through clinical experience that learners are prepared to interact with patients in a natural environment and to become competent independent practitioners (Helgesen, Gregersen & Roos 2016). According to the South African Nursing Council (SANC) education and training standards, practical hours should not be less than 60% of the total duration of the course. This means that clinical practice hours should be more than the theoretical hours (Nursing Act 33, 2005).

In South Africa, learner nurses registered for a 4-year programme are expected to complete a minimum of 4000 h clinical training (R425 of the Nursing Act 50 of 1978). In this study, a learner nurse is a candidate registered in the R425 training programme leading to registration as a nurse (general, psychiatric and community) and midwife. Clinical learning experiences prepare learner nurses to ‘know’ and to ‘perform’ nursing skills. Moreover, Rajeswaran (2016) states that students placed in clinical areas are expected to use their critical thinking skills in solving nursing dilemmas. Soler et al. (2021) argues that the qualities and the art of being a professional nurse are not inherent in the individual but are acquired mainly through clinical experience. When learners enter the nursing profession, they are expected to integrate theory and practice and fit into the multidisciplinary health team. Cowen, Hubbard and Hancock (2018) observe that some learners are excited while others are nervous in anticipation of the clinical experience.

The Real Academia Española (2020) defines expectation as the hope or possibility of achieving something. Soler et al. (2021) argues that for nursing students, expectations could be equated to the learner nurses’ motivation to apply nursing care acquired through theoretical knowledge. In this study, expectations are defined as nursing students’ aspirations and the will to provide care to patients through the application of theory, without fear or insecurities.

On the one hand, clinical experiences are regarded as stressful and anxiety-producing aspects of the training of learner nurses. Suarez-Garcia et al. (2018) point out that during first clinical placements, stressors could include concern about harming patients, lack of necessary skills and fear of being harmed. Wang, Lee and Espin (2019) add that these stressors could further have negative impact on learning, performance and the well-being of learners. On the other hand, despite their anxiety, learners can also be enthusiastic to practise and implement their theoretical knowledge. Their expectations are usually driven by the desire to join the profession. Lovrić et al. (2017) argue that learner nurses’ expectations influence their emotional response and their thinking ability, which will eventually direct their attention and motivation and affect their academic performance.

The authors further add that learner nurses’ expectations are often directed towards performing well in their theory and being competent in nursing practice. Thus, learners expect to provide high quality nursing care and to pass both the theoretical and practical examinations.

Learners’ expectations have been investigated in many countries. In Saudi Arabia, Allari and Farag (2017) found that learners expected clinical learning areas to provide effective learning and a supportive environment. The same expectations of a conducive and supportive clinical learning areas were shared by nursing students in Spain, who indicated that the clinical learning areas’ staff should be supportive, empathetic and approachable and should genuinely care about their learning (Soler et al. 2021). Integrating theory into practice is important and this can be carried out if clinical areas are supportive of learners. The expectations of learner nurses are often not met in clinical learning areas.

### Problem statement

Learner nurses sometimes feel that the clinical learning area staff are not supporting them to meet their learning expectations, and Ten Hoeve et al. (2017) indicate that when learners’ expectations are not met, learners become disappointed, resulting in attrition. Incidences of staff being abusive in their supervisory role and creating an unwelcoming environment have been reported in clinical areas. Chan et al. (2019) suggest that this causes learner disappointment, demotivation and attrition. The researcher, as a nurse educator, often engages with learners after each clinical placement to listen to their experiences and allows them to reflect on their expectations. The researcher’s assumption is that some learners had negative experiences in the clinical areas because of bad treatment. The researcher also assumed that there was no respect and teaching of common conditions in the units, which led learners to feel that there is no integration of theory and practice. Learners had different expectations; the majority felt that their expectations were not met, whereas few indicated that they were met.

### Theoretical framework

The theoretical framework that underpins the study is Benner’s novice to expert model (1984), which describes how learner nurses develop comprehension of patient care through theoretical training and experiential learning (Ozdemir 2019). The model has the following five stages: the novice, advanced beginner, competent, proficient and expert phases. In the novice stage, nursing education institutions provide students with the theory of nursing. Thomas and Kellgren (2017) indicate that this is the first level of training, where simulations are carried out to expose learners to situations they will encounter in the clinical learning areas. At this stage, learners are enthusiastic about joining the profession but nervous at the same time. In this study, these are learners at the first level of training who have no experience in clinical practice, and they must learn the theory provided from the nursing education institutions. The second stage of advanced beginner is when the learner nurses first go to the clinical learning areas, without adequate information regarding individual and personalised patient care. They only have the theoretical knowledge from the nursing education institutions. Thus, learners are only involved in routine work and are likely to be stressed by patients with complex conditions and heavy workloads. At this stage, learners learn by trial and error and expect support and guidance from the senior staff. Learners at the first and second level of study are at this stage of the model. Ozdemir (2019) recommends that at this stage, professional nurses and senior learners should assist the advanced beginner to integrate theory and practice.

In the competent stage, learners gain competency in planning patient care. They are less anxious and perform well with minimal mistakes. At this stage, it is important to evaluate the performance of the learner, as this allows them to introspect and improve on their performance. Learner nurses in the third and fourth year of study are at this stage. They are able to perform most skills in clinical practice. They still practise under the supervision of the professional nurse at a minimal scale. Ozdemir (2019) points out that at the end of third year, competency could be evaluated through practical examinations. In the fourth stage of being proficient or competent, learner nurses become more confident in the implementation of patients’ individualised nursing plans. At this stage, learner nurses can mentor and coach other junior learners, and they can assume leadership roles in hospital management or professional organisations. Thomas and Kellgren (2017) elaborate that at this stage, learners spend less time and energy in thinking and planning as they simply know what needs to be performed.

The expert stage is the final stage in Benner’s novice to expert model. Learner nurses in their fourth year have reached this stage. They are more competent and confident and are experts. They feel free to practise without causing any harm to patients, as they are skilled. At this stage, learner nurses make their own critical decisions in patient- and unit-related matters because they would have achieved the desired competency and level of practice that is expected of them. On completion of training, the learner can integrate theory and practice and is competent in clinical practice.

### Purpose

This study sought to understand learner nurses’ expectations of the clinical learning areas during clinical placement.

### Objectives

The study’s objectives are to explore and describe the expectations of learner nurses during clinical placement in the clinical learning areas.

## Methodology

A qualitative, descriptive and contextual design was used to explore the expectations of learner nurses from the first year to the fourth year of training (Polit & Beck 2018). Aspers and Corte (2019) define qualitative research as an iterative process where improved understanding of the phenomenon being studied is obtained through scientific gathering of information. Qualitative researchers study phenomena in their natural settings, attempting to make sense of or to interpret phenomena in terms of the meanings attached to them (Aspers & Corte 2019). The qualitative approach was appropriate for this study because the researcher wanted first-hand information from participants. Participants expressed their feelings and shared expectations of the clinical learning areas. The descriptive design was chosen so that participants could describe their expectations of clinical learning areas. The contextual design gave the participants the opportunity to express those expectations within the context of the clinical areas, which they are allocated for clinical learning experience.

### Setting

Four clinical learning areas in the Limpopo province were purposely sampled. The four clinical areas were tertiary, regional and district health settings. The researcher, who is the first author, collected data from settings of care at different levels because the number of professional nurses and the available resources at these clinical areas differ. Tertiary and regional hospitals have many professional nurses because they are referral hospitals. Patients who utilise the services in referral hospitals are more than those who use district hospitals. Learners from the colleges and universities who train for the R425 programme are allocated these referral hospitals.

The Limpopo province is predominantly rural and situated in the northern part of South Africa, bordering Mozambique, Zimbabwe and Botswana. The languages most spoken in the province are Sepedi, Tshivenda and Xitsonga. The capital city of the province is Polokwane where one tertiary hospital was sampled.

### Sample

Nonprobability purposive sampling was used to sample learners in the R425 programme from the sampled universities and nursing colleges in the Limpopo province. The inclusion criteria for selection were learner nurses training for the R425 programme at colleges and universities in the province. These are learners who were allocated for clinical placement at the sampled clinical learning areas and were willing to participate in the study. The research population constituted both male and female learners. Learners in all levels of training were included in the study. In the focus group discussions (FGDs), level one had 6 participants, level two had 7, level three had 12 and level four had 15. Learners in levels three and four of the programme were the majority of the participants, so these two levels had two focus groups each. Ten participants were male and 30 were female. The exclusion criteria were learners in other nursing programmes (for example, those in post-basic courses, the Bridging course and Midwifery Science). They were excluded from the study because they had previous training experiences, and the researcher assumed that their expectations would differ from those who were receiving training for the first time.

### Data collection

Data were collected through focus group discussions (FGDs). Learner nurses were recruited after obtaining permission from selected nursing education institutions. Learners who wanted to participate were briefed about the purpose of the study and the researcher’s expectations. Invitation letters to participate were given to willing participants. Six FGDs were conducted; each group had 6–8 members. Participants were grouped according to their level of training, as the researcher assumed that expectations might differ depending on the level of study. In each FGD, there was a moderator to ensure that there was no dominance and wastage of time and that the discussions ran smoothly. For data collection, arrangements were made with participants to meet at a time convenient for them. The researcher was the main research instrument as she conducted the interviews in the FGDs. Participants were asked the following questions: (1) when you are in the clinical areas, what are your expectations? (2) Are those expectations met? The FGDs were audio-recorded and later transcribed verbatim. During data collection, field notes on important points were taken and the expressions of the participants observed. Probing was carried out to seek clarity and gain more insight on the mentioned aspects. The interviews were performed in the clinical learning areas to avoid disturbing classes during May to November 2019. Each FGDs lasted for 45–60 min.

### Data analysis

Data were analysed through Tesch’s (1992) open-coding method, which include the following steps: (1) to get a sense of the whole, the researcher read through all the transcripts to capture the entire meaning of the information; (2) one most interesting transcript was picked and read through, making reflective and marginal remarks as meaning came to the fore; (3) similar topics were clustered together using columns and colour shades, clustering according to major topics, unique topics and leftovers; (4) topics were abbreviated and the coded topics were organised into small categories of information; (5) the descriptive topics were turned into themes and sub-themes where the total categories were reduced by grouping topics that related to each other; (6) the categorised themes were abbreviated to ensure that all categories were included; (7) similar data material were assembled and a preliminary analysis was carried out; and (8) all the existing data were coded to ensure that nothing was missed (Creswell & Poth 2016). The assistance of two researchers with knowledge and experience on qualitative research was sought to assist in the development of themes and sub-themes.

### Measures to ensure trustworthiness

Trustworthiness refers to the degree of confidence qualitative researchers have in their data, using the criteria of credibility, transferability, dependability and confirmability (Polit & Beck 2017). Credibility was ensured through prolonged engagement, which encouraged the researcher and the participants to develop trust towards each other, as this is significant in qualitative research. The researcher gave participants adequate time to express their expectations without interruptions. Data were collected over a period of 7 months, leading to more engagements with participants. Member checking was carried out where the researcher met with the participants after data collection. Preliminary findings of the researcher were discussed with the participants to ensure that the collected data were true reflections of what participants indicated. Member checking was performed at one of the sampled clinical areas. Peer review was performed through consultation with two experienced qualitative researchers who gave inputs and guidance during data collection and processing. Transferability was ensured through rigorous methodology where the sampling and data collection methods were thoroughly defined, described and implemented. The research sample included learners in all levels of study to ensure that expectations were explored as per level of study. Focus group discussions were used to collect data, as expectations could be shared depending on the level of study. To ensure dependability, data were analysed by two independent researchers, one being an independent coder who compared the results, and in this instance the results were mostly similar. All voice recordings, transcripts and field notes were stored in case an audit trail is needed. Authenticity was ensured through member checks, where the results were discussed with participants to ensure that the information and the interpretation thereof was correct.

### Ethical considerations

Ethical clearance to conduct this study was obtained from the University of Venda Research Ethics Committee (reference number: SHS/19/PDC/04/1103) on 25 March 2019. Letters requesting to collect data were sent to the Department of Health in the province, district and sampled institutions where permissions to conduct the study was granted. With the permission of the nursing service managers, learners were addressed in the provided venues at different institutions. Participants were told about the purpose of the study, their right to decline participation, the right to privacy, the right to be free from harm and the right to withdraw from participation without any penalty. Written consent was obtained from the participants before the study was conducted.

## Results

The findings of this study are based on the analysis of students’ responses to the questions asked. Main themes, themes and sub-themes emerged from the study. The three main themes that emerged were expectations in relations to the milieu in the clinical learning areas, learning and self-actualisation. Table 1 summarises the themes and sub-themes that emerged.

**TABLE 1:** Themes and sub-themes.

No.	Themes	Sub-themes
1.	Expectations regarding the milieu in the clinical learning	Poor support from the clinical staff
		Poor interpersonal relationships with clinical staff
		Professional nurses not being exemplary role models at all times
2.	Learning needs expectations	Lack of correlation between theory and practice
		To be competent in practice
		Being evaluated on their performance
3.	Self-actualisation expectations	Being mentored
		To be independent practitioners

### Theme 1: Expectations regarding the milieu in the clinical learning areas

Most learners in the first and second year of study indicated that when they are in clinical learning areas, they expect professional nurses to offer them support and guide them during the execution of their allocated responsibilities. This is the beginner’s or novice stage of learning where learners are dependent on trained staff for practical knowledge. Although the support from professional nurses was there, it was not enough as some professional nurses were not interested in guiding and mentoring learners. Participants also stated that the relationships with some clinical staff members were not good. Participants felt that the welcoming and interpersonal relations with experienced staff were not good.

#### Sub-theme 1.1: Poor support from the clinical staff

Learners said that trained staff should offer them support during clinical placement, such as being in the units and receiving assistance when there are challenges in providing care, and the experienced staff should care about learners’ well-being. Some participants indicated that they received support, whereas others indicated a lack of support from the clinical staff. In relation to the support from professional nurses, the following transcripts support this sub-theme:

‘In the clinical areas, I expect that the professional nurses should guide me, and when they are busy with providing care, they should explain what they are doing and what are the complications. Often, the professional nurses support us; they teach and tell you to ask if you don’t understand.’ (Participant D, group 1, first level of study, undisclosed gender, undisclosed years)

‘Professional nurses in the wards are not there for support all the time. Sometimes when you ask them about something which you are not completely sure and ask for their assistance, they indicate that they are busy and don’t have time to (Participant N, group 2, second level of study, male, 20 years)

#### Sub-theme 1.2: Poor interpersonal relationship with clinical staff

Participants’ expectations were that they should be welcomed warmly and in a manner that would make them feel at ease. They indicated that they need to be orientated by the staff in the placement areas and to be shown the physical layout of the unit. They expected to be treated with respect and not to be bullied and assumed that they would be less stressed by their clinical placement as a result. Participants indicated that a significant number of clinical staff interacted with them properly and only a few were unpleasant.

Leaner nurses cited lack of orientation and feeling unwelcomed. They felt that professional nurses do not orient them properly. This view is expressed by a first-year learner:

‘My expectation is that when I am in the clinical area, the staff should welcome me and orientate me to the unit so that I don’t get lost and waste time when I have to go and fetch something, maybe, from the storeroom. Again, if one is orientated and warmly welcomed, the stress that you had before decreases. Some professional nurses just shout at us without any reason.’ (Participant F, group 1, first level of study, female, 18 years)

Some participants stated that staff treat them differently from other team members. They expect to be addressed by their names and not just as students. Learner nurses in the fourth year considered themselves professional nurses in the making and wanted to be treated as other members in the clinical areas. They said that professional nurses do not treat them with respect, as they treat other qualified workers in the clinical areas. This is indicated in the following quote:

‘Staff in the wards treat us differently from how they treat each other. When they talk to us, they are commanding and do not even call us by our names. They refer to us as “you students” as if we are generalised.’ (Participant A, group 6, fourth level of study, male, 23 years)

#### Sub-theme 1.3: Professional nurses not acting as exemplary role models at all times

Participants wanted professional nurses to be exemplary all the time. A role model is a person whose behaviour and conduct are according to the prescripts of the law and is worthy of emulation; a role model is a positive example of a member of a profession (Jack, Hamshire & Chambers 2017). When learner nurses join the profession, the person whom they aspire to be is a professional nurse, and thus student nurses emulate professional nurses. Participants said most professional nurses acted as good role models.

Third- and fourth-year students already know and have experience of clinical activities and responsibilities and can differentiate the acceptable and unacceptable conduct by staff members. At this level, learners have an idea of an ideal professional nurse. These views are expressed:

‘In the clinical placements, I expect professional nurses to be exemplary role models so that as students we can copy what they do. They are our seniors, and we want to be like them one day; therefore, they should only do what is right. They must be competent and not shout at patients when they are working.’ (Participant C, group 4, third level of study, female, 20 years)

Incidents where professional nurses violated professional ethics were reported. This view is expressed by a number of participants:

‘When I am in the clinical learning areas, my expectation is that professional nurses, as seniors, should uphold the ethical standards of the profession. They need to ensure that the language they use when speaking to patients is of respect and ensure dignity. Some of them do not always show sympathy and empathy. In most instances, they are rude to patients and I feel it is very wrong.’ (Participant L, group 5, fourth level of study, female, 21 years)

‘Even though we expect professional nurses to be our role models, at times they act in a manner that we can see that it is not correct.’ (Participant A, group 3, third level of study, male, 21 years)

After being asked to elaborate, Participant A continued:

‘Yes. Some shout at patients and do not respond when patients call them, and I feel that is wrong. Some take long tea breaks and lunch. We usually just keep quiet but we don’t do what they do.’ (Participant A, group 3, third level of study, male, 21 years)

### Theme 2: Learning needs expectations

Learner nurses indicated that when they go for clinical placement, they expect their learning needs to be met. Some learners felt that their expectations were met, whereas others felt otherwise. Three sub-themes emerged from this theme and these are as follows.

#### Sub-theme 2.1: The lack of correlation between theory and practice expectations

Most learners viewed clinical areas as a place where they would integrate theory and practice. They expected to practice what they would have learned in class in real-life situations.

Learner nurses in the first and second year of study do not have a great deal of experience in clinical practice, although they may have theoretical knowledge. Therefore, their theoretical knowledge should be tested during practice. It is in the clinical learning areas where the integration of the two components of learning take place. Participant A shows understanding of this in the following words:

‘We go to the clinical areas after being taught about the different procedures at the simulation lab and also in class, and when we are in the clinical area, I expect that I should be able to practise what our lecturers taught us.’ (Participant A, group 1, first level of study, gender undisclosed, years undisclosed)

Experienced learners, such as third- and fourth-year learners, know clinical procedures. However, some reported witnessing procedures in clinical areas that contradict their theoretical education from their institutions. In this regard, Participant E said:

‘Although professional nurses do things differently from what was taught, we try to do what was taught.’ (Participant E, group 3, third level of study, gender undisclosed, years undisclosed)

On probing about what the participant meant by saying professional nurses do things differently, the participant explained:

‘When they do procedures, they often do not follow the methods, as they say that it is time consuming, and they need to finish the procedures quickly and do other tasks.’ (Participant E, group 3, third level of study, gender undisclosed, years undisclosed)

#### Sub-theme 2.2: Being competent in practice expectations

Learner nurses expect to achieve their goals and those set by their training institution. Most students indicated having a fulfilling feeling when they have executed a task to the best of their ability, because competency gave them confidence to execute more challenging tasks. Learners indicated that at the end of each placement, they expect to be competent in the procedures that are performed in different units.

Learner nurses in their final year wanted to be competent in performing clinical procedures. This accords with Benner’s model, where competency was important as students increasingly become aware that they would soon be professional nurses. In this regard, Participant M said:

‘My expectation is that at the end of each allocation, I must be able to perform the procedures that are carried out in each ward; for example, when I am allocated surgical ward, at the end of the allocation I expect to be able to prepare patients for surgery and to care for wounds.’ (Participant M, group 5, fourth level of study, female, 22 years)

Further discussions were about patients’ care and satisfaction. Learner nurses indicated that they need to meet patients’ needs to the latter’s satisfaction:

‘I expect to be able to provide care to patients and do it well so that the patient healed, and I should feel happy about what I did. I want to be competent in whatever I do to patients.’ (Participant S, group 6, fourth level of study, female, 24 years)

Competency was also an expectation from level two learners, as at their level of training, they have been allocated for clinical learning on several occasions and have acquired certain skills. The sub-theme is supported by the following quote from third-level Participant R, who said:

‘My expectation is that I should be competent in providing care to patients, as I have already been taught about the procedures and I have demonstrated them back. I expect that when I perform the procedures, I should do it to the best of my ability and make the patient to recover.’ (Participant R, group 4, third level of study, female, 20 years)

#### Sub-theme 2.3: Being evaluated on performance expectations

Participants expected professional nurses to evaluate their performance after clinical placement and to let them know whether their performance was satisfactory and where they should improve. Some stated that evaluation is not performed, and this frustrates them as they leave the clinical areas with their performance and conduct not having been evaluated. Learner nurses in levels one and two wanted their performance in the clinical learning areas to be evaluated because they are new in training and depend on professional nurses to guide them.

The following responses support the given observations:

‘My expectation is that at the end of each placement, the professional nurses should sit down with each one of us towards the end of the placement in that unit and give feedback on the performance so that the learner should know if she was doing well or not. … Professional nurses need to tell us if we were doing procedures correctly and coming on time to the clinical areas.’ (Participant F, group 1, first level of study, female, 19 years)

‘They should also evaluate us on our conduct and behaviour so that those who dodge can improve their ways. They often just keep quiet and let us go without saying anything.’ (Participant S, group 1, first level of study, male, 18 years)

Learners in their second and third year of study indicated that they expected professional nurses to evaluate them in order for them to be sure that they were on the right track. They also indicated that evaluation would make them feel happy, as they would know that they are capable. In support of the sub-theme:

‘My expectation is that at the end of the allocation, professional nurses should give me feedback, as that will encourage me to work harder. It will also make me happy knowing that I am competent in providing care to patients.’ (Participant J, group 2, second level of study, female, 19 years)

‘I expect professional nurses to sit down with us at the end of each shift to tell us where we did good and where we should improve our performance. This is important to me because if I just work without any feedback, I think nobody is watching and no one cares. To me, feedback is important, as it gives me a direction of where I am going.’ (Participant Z, group 4, third level of study, male, 20 years)

### Theme 3: Self-actualisation expectations

Learner nurses at the fourth level of training are expected to be given the opportunity to practise as professional nurses in the making, because they are in the expert stage of Benner’s model. They wanted to be mentored to be independent practitioners to become relaxed and confident in clinical practice.

#### Sub-theme 3.1: Being mentored

Participants who were in their final year of study expected to be assigned professional nurses as mentors because they wanted to assume more responsible roles. They stated that being mentored and taking more responsible roles would assist them when they become professional nurses:

‘At this level, I expect the professional nurses to mentor me. They should allocate me with one of the professional nurses in daily activities so that I learn what professional nurses do in their day-to-day responsibilities.’ (Participant P, group 5, fourth level of study, female, 22 years)

#### Sub-theme 3.2: To be independent practitioners

Learners at the fourth level stated that during clinical placement, they expect to be independent practitioners, as they would be close to completing their training. According to Benner’s model, at this level learners are competent and can practise independently. Fourth-year learners wanted to be allocated professional nurses’ tasks because they felt confident and competent. These comments are supported by the Participant G’s response:

‘Fourth-level students should be given the responsibilities given to professional nurses like writing off duties, delegation and ordering of supplies. As we shall be completing, we need to complete with those skills, as they are necessary in the role of a professional nurse.’ (Participant G, group 6, fourth level of study, female, 23 years)

## Discussion

This study explored expectations of learner nurses during clinical placement. Learners in the first and second year of study expected competency in practice, whereas those in their third and fourth year wanted independence and self-actualisation because they were close to completing their training. The first expectation of their training journey was to integrate theory and practice. The integration of theory and practice begins early in learners’ training, laying the foundation for a lifetime of continuous learning (Gassas 2021). Failure to integrate theory and practice can lead to a situation where learners fail to adjust to the profession. This sentiment is shared by Safazadeh et al. (2018), who indicate that the cause of theory and practice gap in clinical learning is because of learner nurses’ attitude towards the profession, the engaging nature of theoretical learning and the existence of a learning atmosphere in the clinical environment. A learner who is not self-motivated might find it difficult to learn and merge theory and practice (Mafumo, Tshililo & Luhalima 2022). Learner nurses had opinions that the clinical learning areas should be supportive and conducive to learning by creating an environment conducive to learning and thereby helping the learners to integrate theory and practice.

Integration of theory and practice in the clinical areas is a challenge, as indicated by study participants. What creates confusion is that procedures are sometimes performed differently in the clinical areas compared with what learners are taught at educational institutions. Similarly, Soler et al. (2021) found that learners were challenged in integrating theory and practice because in educational institutions procedures are simulated, whereas in the clinical area, learners interact with real people who have feelings and emotions. Mariyanti and Yeo (2019) states that another challenge in integrating theory and practice is that the clinical learning environment is unsupportive and does not promote teaching and learning. If the staff in the clinical learning areas offer guidance and support to learners, this could lead to learners improving their communication and interaction skills with patients. Learners indicated that in some units there is only routine work, and therefore they find it difficult to integrate theoretical knowledge because they only do repetitive work. Bazrafkan and Najafi Kalyani (2018) also found that routine work could negatively influence clinical learning, as learners find it difficult to integrate theory and practice in an environment where routine work is practised.

Participants desired to be competent in providing care, and they said competency is providing the expected care to the patients. Nehrir et al. (2016) argues that competency is based on the theoretical knowledge that students would have acquired. Competency is critical in nursing to prevent medicolegal hazards and because once something goes wrong, it cannot be reversed. The lives of patients could be in danger. Competency can be evaluated through feedback and supporting students during clinical experience.

Participants had expectations regarding evaluation of their performance after clinical placement. Adamson et al. (2018) point out that professional nurses should give honest, accurate and constructive feedback to learner nurses, as this has a positive influence on the learning experience. Evaluation is significant to learner nurses as it gives them time to reflect and introspect on their performance. Evaluation would likely lead to satisfied performance and encourage learners to do more. On the other hand, evaluation may encourage the unsatisfied learners to want to improve their performance. This is also a point made by Cant, Ryan and Cooper (2021) when they say strong satisfaction motivates learners to put in more effort and increases positive attitudes towards learning.

Learner nurses stated that the environment in the clinical areas is important to their learning. Lovrić et al. (2017) indicate that learners expect the milieu in clinical learning areas to be supportive to their training and to be relaxed, with good interpersonal attributes. Participants expected clinical staff to be supportive by using teachable moments when there are rare conditions in the units. Lamont et al. (2015) also argues that the relationship between the learners and clinical staff is important, as it may either promote or hinder learning. Students expected to be treated with respect and addressed properly. Birks et al. (2018) indicate that bullying and mistreatment of nursing students is a common phenomenon worldwide. Learners reported being bullied not only by nursing staff but also by other members of the health team. Minton and Birks (2019) state that bullying of learner nurses and subjection to uncivil behaviours during clinical placement have negative consequences, and these may have physical, psychological and financial implications, which might lead them to leave the nursing profession. Panda (2021) concurs that bullying is bad for learning and should be avoided.

Role modelling by professional nurses was emphasised by third- and final-year students. Exemplary role modelling plays an important in instilling professional values to learners. It is through role modelling that new members are immersed into the profession. Bussard and Lawrence (2019) state that Bandura’s social learning theory indicates that people learn through observing others. De Swardt, Van Rensburg and Oosthuizen (2017) found that students considered nurses in the clinical field to be the most influential role models in shaping their clinical practice and their socialisation process. Therefore, it is important that professional nurses consistently act as exemplary role models.

Lovrić et al. (2017) observe that learner nurses’ expectations change over time. Final-year students expect to be prepared to become independent and competent practitioners, because learners are aware of their increased responsibility. They also wanted mentors who would take their hand and lead them until they complete their training.

### Limitations

This study was conducted in the predominantly rural Limpopo province, and an urban setting may yield different results. The study population was learner nurses registered for the R425 programme. It is likely that if there were learners registered in other undergraduate and post-basic nursing programmes, findings could have been different. Moreover, the researcher did not consider all variables that might have influenced research results, such as the fact that expectations might differ depending on the different settings of different levels (tertiary, regional and district hospitals).

### Recommendations

#### Recommendations to nursing practice

The nature of clinical areas can be stressful and intimidating to learners, especially during the first years of training, and it is therefore crucial that professional nurses offer support to learners. As stated in Benner’s model, learner nurses who are in the first level of study and going for clinical learning are in the advanced beginner stage. During initial placements, students often have mixed feelings, they are often anxious but eager to learn at the same time, as they will be going for clinical learning for the first time; therefore, they need supportive leaders in the clinical areas. They need to be orientated about the physical layout and all the activities in the clinical learning areas. They also need to be to be guided, as they are still finding themselves. Learner nurses in the competent phase, which is usually in the second and third level of training, are expected to be competent in practice. Competency is evaluated by the professional nurses who have knowledge and are competent themselves. During and at the end of each clinical placement, professional nurses need to give learner nurses feedback so that they can know if they are competent or need to improve on their performance. Final-year students are in the expert stage in Benner’s model, and they should be mentored by other professional nurses so that they are oriented into their future responsibilities when they finish their training. They should also be given challenging tasks so that they acquire critical thinking and decision-making skills in the units.

#### Recommendations to nursing education

Each unit in the clinical learning areas should have learning objectives so that professional nurses can use them to evaluate learner performance. Nurse educators who do clinical accompaniment should support learners and ensure that challenges in the clinical learning areas are identified and corrected.

#### Recommendations to nursing research

The clinical learning areas are grounds for nursing practice because learner nurses acquire their skills in those areas. Research on supporting professional nurses in nurturing and meeting learners’ expectations should be carried out so that future professional nurses are equipped with the skills and resources that will enable them to support novice learners.

## Conclusion

Learner nurses perceive clinical learning areas to be significant in their training. When they are allocated to clinical learning areas, their expectations are driven by their motivation to achieve their desired goal of integrating theory acquired from nursing educational institutions into practice. They want to be competent in providing care to patients. They also expect clinical learning areas to be welcoming with a conducive learning environment. Learners who were completing their training wanted to be recognised as future nurse practitioners by being given tasks that would equip them with the skills needed to become independent and competent practitioners.
